# Depletion of the p43 Mitochondrial T3 Receptor Increases Sertoli Cell Proliferation in Mice

**DOI:** 10.1371/journal.pone.0074015

**Published:** 2013-09-09

**Authors:** Betty Fumel, Stéphanie Roy, Sophie Fouchécourt, Gabriel Livera, Anne-Simone Parent, François Casas, Florian Guillou

**Affiliations:** 1 INRA, UMR85 Physiologie de la Reproduction et des Comportements, Nouzilly, France; 2 CNRS, UMR7247 Physiologie de la Reproduction et des Comportements, Nouzilly, France; 3 Université François Rabelais de Tours, Tours, France; 4 IFCE, Nouzilly, France; 5 Université Paris Diderot, Sorbonne Paris Cité, INSERM U967, CEA/DSV/iRCM/SCSR Laboratoire de Développement des Gonades, Fontenay-Aux-Roses, France; 6 Developmental Neuroendocrinology Unit, GIGA Neurosciences, University of Liège, CHU Sart Tilman, Liège, Belgium; 7 INRA, UMR 866 Dynamique Musculaire et métabolisme, Montpellier, France; 8 Université de Montpellier 1 et 2, Montpellier, France; Clermont-Ferrand Univ., France

## Abstract

Among T3 receptors, TRα1 is ubiquitous and its deletion or a specific expression of a dominant-negative TRα1 isoform in Sertoli cell leads to an increase in testis weight and sperm production. The identification of a 43-kDa truncated form of the nuclear receptor TRα1 (p43) in the mitochondrial matrix led us to test the hypothesis that this mitochondrial transcription factor could regulate Sertoli cell proliferation. Here we report that p43 depletion in mice increases testis weight and sperm reserve. In addition, we found that p43 deletion increases Sertoli cell proliferation in postnatal testis at 3 days of development. Electron microscopy studies evidence an alteration of mitochondrial morphology observed specifically in Sertoli cells of p43−/− mice. Moreover, gene expression studies indicate that the lack of p43 in testis induced an alteration of the mitochondrial-nuclear cross-talk. In particular, the up-regulation of Cdk4 and c-myc pathway in p43−/− probably explain the extended proliferation recorded in Sertoli cells of these mice. Our finding suggests that T3 limits post-natal Sertoli cell proliferation mainly through its mitochondrial T3 receptor p43.

## Introduction

Sertoli cells (SC) are essential in spermatogenesis. It is well established that the number of SC conditions the efficiency of spermatogenesis as one SC can only support a limited number of germ cells [1]. SC number is defined during the foetal and pre-pubertal periods. The SC proliferation arrest mainly depends on thyroid hormone T3. In rodents, during the post natal period, the arrest of SC proliferation and entry into differentiation is correlated with a peak of T3 blood level [2]. Neonatal hypothyroidism in mice and rats leads to an increase in the weight of adult testis and epididymis due to an increase in the daily sperm production [3]–[8].

For a while, it was assumed that thyroid hormone acts exclusively through nuclear receptors: TRα1, TRα2, TRβ1, TRβ2 encoded by the *THRA* and *THRB* genes [9]. Among T3 receptors, TRα1 is ubiquitous and it was previously described that its total deletion in mice leads to an increase in testis weight and sperm production [10]. Moreover, a specific expression of dominant-negative TRα1 isoform in SC exhibited similar phenotypic features to the TRα1 knock-out mice: heavier testicular weight and higher sperm reserve [11]. In 1995, a 43-kDa truncated form of the nuclear receptor TRα1 was identified in the mitochondrial matrix (p43) [12]. This protein is synthesized by the use of internal initiation site of translation occuring in the TRα1 transcript. The p43 is a mitochondrial T3 receptor ubiquitously expressed that stimulates mitochondrial transcription and protein synthesis in the presence of T3 [13]. The physiological importance of p43 was recently revealed by the use of mice overexpressing or lacking this mitochondrial receptor. In particular, these works establish that p43 receptor strongly affects muscle mass and the metabolic and contractile features of myofibers [14]–[16]. In addition, p43 was found to regulate insulin secretion and glucose homeostasis [17].

In this work, we focused our study on testis because the presence of high-affinity binding sites for T3 has been reported in rat Sertoli cell mitochondria [18]. Here we report that p43 specific depletion in mice increases testis weight and sperm reserve. In addition, we found that p43 deletion increases Sertoli cell proliferation in postnatal testis at 3 days of development. Moreover, gene expression studies provided some molecular mechanisms underlying the ability of T3 to arrest Sertoli cell proliferation. In particular, c-myc and Cdk4 could be the central targets of the mitochondrial-nuclear crosstalk, controlling the proliferation of Sertoli cells. In summary, we demonstrate that the T3 limits post-natal Sertoli cell proliferation in part through its mitochondrial T3 receptor p43.

## Materials and Methods

### Animals

All animal experiments were performed according to European directives (86/609/CEE) and approved by the Comité d’Ethique en matière d’Expérimentation Animale : Région Languedoc-Rousillon. Our animal studies were conducted in accordance with guidelines for the care and use of laboratory animals issued by the french Ministry of Agriculture.

Mice were fed a standard laboratory diet and tap water ad libitum. The p43−/− mice lacking specifically the mitochondrial T3 receptor were generated by François Casas as described previously [17]. To only inhibit p43 translation from TR1 mRNA, we deleted the internal translation start in exon 3 using site directed PCR mutagenesis to change the methionine codon at position 109 to a leucine codon. Targeting construct was electroporated into 129SV embryonic stem (ES) cells. We generated chimeras by blastocyst injection of ES cells and obtained germline transmissionof the mutated allele. We mated them with C57BL/6 females. Offspring inheriting the p43-mutated allele were intercrossed to generate the p43−/− line. Homozygote p43−/− mice born from the 3 independent ES cell clones were viable, fertile, and appeared normal. All the mice used in these studies were back-crossed at least 10 times into the C57BL/6 background.

The colony was generated by crossing p43−/− mice with wild-type (WT) C57BL/6 breeders and generated future generation of WT controls.

### Determination of Testis Weight and Sperm Reserve

Adult p43−/− and their respective control mice were sacrificed and one testis was weighed and then frozen for sperm reserve determination as described previously [19]. Briefly, testis were disrupted in 3 ml of L15 medium (Gibco-Invitrogen) in a glass potter before sonication for 30 seconds. Remaining sperm nuclei were counted using hemocytometry. These nuclei contain spermatozoa and elongating spermatid nuclei (stages II-VII), and their number defines the testicular sperm reserve [8]. The testicular sperm reserve/mg was obtained after dividing whole testicular sperm reserve by the weight of the testis.

### Fertility Test

Each male (n = 30 per genotype) was mated with two primiparous C57/black6 females; birth date were noted to detect a putative delay in mating, and pups were counted at birth.

### Plasma Testosterone

Mice were treated with an intraperitoneal injection of 15IU/animal of human chorionic gonadotropin (hCG) (Chorulon). Blood was collected before (basal level) and two hours after this injection (stimulated level). The plasma was stored at −20°C until tritium-based testosterone competitive radio-immunoassays were carried out as described previously [20]. The sensitivity of the assay was 0.125 ng/ml and the intra-assay coefficient of variation was 7.5%. Briefly, samples (two dilutions per sample) or testosterone dilutions (to determine the range) were incubated for 1h at 40°C (Buffer: 0.1 M phosphate buffer 0.1% gelatin) with tritiated testosterone plus the anti-testosterone antibody. A secondary antibody was then added and the mixtures incubated for one night at 4°C. Immuno-precipitation was then performed with PEG (PolyEthyleneGlycol) 4000 and radioactivity was counted (Packard C2900 TriCarb).

### Serum FSH Levels

Serum FSH levels were determined in a volume of 100 µl using a double Ab method and a RIA kit (rFSH RIA), kindly supplied by the National Institutes of Health (Dr. A. F. Parlow, National Hormone and Peptide Program, Torrance, CA). Rat FSH antigen (NIDDK-rFSH-I) was labeled with ^125^I by the chloramine-T method and the hormone concentration was expressed using the rat FSH reference preparation (NIDDK-rFSH-RP-2) as standard. Intraassay and interassay coefficients were less than 7 and 10%, respectively. The sensitivity of the assay was 0.125 ng/100 µl.

### Testis Histology

Testis histology (seminiferous tubule organization) was analyzed after fixing in Bouin’s fluid and embedding in paraffin. Sections (4 µm) were stained with haematoxylin for microscopic observation.

### Determination of SC Proliferation Index in Vivo

p43−/− and WT mice at post-natal day 3 (P3) and 10 (P10) were injected with 50 µg/g (of body weight) of 5-bromo-2-deoxyuridine (BrdU) (Sigma) 3 hours before sacrifice and BrdU was incorporated in proliferating cells. Testis were fixed in Bouin’s fluid, embedded in paraffin and sectioned (4 µm). After antigen retrieval in a boiling sodium citrate buffer (pH 6.0), endogenous peroxidase activity was quenched with H_2_O_2_. BrdU immunodetection was performed using a mouse IgG monoclonal antibody (Roche; 1/200), and revealed using a peroxidase labelled polymer conjugated to goat anti-mouse IgG (Dako) and 3 3′diaminobenzidine chromogen (DAB) (Dako). Negative-controls were processed without the BrdU antibody. After counterstaining with haematoxylin, a total of 1000 proliferating (BrdU stained) and quiescent SC were counted (blindly) per animal using Histolab (GT Vision) analysis software.

### RNA Extraction and Quantitative RT-PCR (Q-PCR) of Testis at P3

Total RNA was isolated from whole testis at P3 using Trizol reagent (Invitrogen) according to the manufacturer’s instructions. To quantify the expression of genes involved in the cell cycle and in mitochondrial activity, we used Profiler PCR Array technology (Qiagen; http://www.sabiosciences.com/PCRArrayPlate.php) as recommended by the manufacturer and used by others [21]. Gene expression levels were normalized using five reference genes in the array: β-glucuronidase, hypoxanthine guanine, heatshock protein 90ab1, glyceraldehyde-3-phosphate dehydrogenase and β-actin. The comparative cycle threshold (Ct) method was used to calculate the relative quantification of gene expression. The fold change (FC), which represents the variation in the level of expression between p43−/−and the control mice, was calculated using the web-based RT2 profiler PCR Array Data Analysis program. A list of differentially expressed genes was identified using a 2-tailed t-test, with a P value <0.05 (t-test) and a mean difference equal to or greater than 2-fold (top right area). To determine JunD, c-myc and β-actin mRNA levels by qRT-PCR, RT were performed using MMLV- RT (Invitrogen) according to the manufacturer’s recommendations. Quantitative RT-PCR reactions were performed using SYBR Green SuperMix (Biorad) according to the manufacturer’s instructions and run in triplicate in an iCycler (Biorad). Specific primers and hybridization temperatures have been previously described [11]. In order to control the differences in RNA concentrations between each sample, the transcript level of each target gene was normalized on the basis of the transcript levels of the constitutive housekeeping gene β-actin (once we had verified that β-actin mRNA levels were not significantly different between samples).

### Western Blotting

Testes at post natal day P3 were sliced and homogenized in 50 µl of lysis buffer (Tris 10 mM pH 7.4, Triton X-100 0,7%). The homogenate (40 µg) were denatured and electrophoresed onto 12% SDS-PAGE and transferred onto a nitrocellulose membrane. Proteins were visualized by enhanced chemiluminescence (32106, Pierce®, Rockford, IL) and quantified with ImageJ® software. Antibodies Cdk4 (sc-260) and β-Actin (sc-81178) were purchased from Santa Cruz Biotechnology.

### Transmission Electron Microscopy

Testis from two different animals per genotype at 5 months of age were collected and fixed in 4% glutaraldehyde in cacodylate buffer 0.1M and further fixed in 1% osmium tetroxide in cacodylate buffer prior to being embedded in Epoxy resin. Sections (70 nm thick) were placed on 200-mesh copper grids, stained with uranyl acetate followed by lead citrate and examined using an electron microscope (CM 10 Philips, Eindhoven, The Netherlands).

### Statistical Analysis

All data are presented as means ± SEM. To compare means between two groups, the Student *t* test, or the Mann-Whitney *U*-test in case of differences in variance (Fisher test), were used.

Other comparisons were performed using a two-way ANOVA followed by the Bonferroni post test. *P*<0.05 was considered significant. Statistical analyses for the interpretation of “Volcano Plots” were proposed by the Web-based RT^2^ profiler PCR Array Data Analysis program.

## Results

### Depletion of the p43 Mitochondrial T3 Receptor Does not Affect the Gene Expression of Other Nuclear Isoforms of TRα

Many isoforms of TRα receptors have been identified. Among them, two nuclear forms (TRα1 and TRα2) and a 43-kDa truncated form of the nuclear receptor TRα1 located in the mitochondrial matrix was previously described [9], [13]. Total deletion of all TRα receptors leads to an increase in testis weight and sperm production. To assess the physiological importance of p43 for testis development, we used mice carrying a specific p43 invalidation obtained by the mutation of the internal site of translation of the mitochondrial protein [16]. As previously described in skeletal muscle [16], we found that TRα1 and TRα2 mRNA expressions in testis are not different in wild type (WT) and p43−/− mice (data not shown).

### The Depletion of p43 Leads to an Increase in Testis Weight and Sperm Reserve

**Figure 1:**
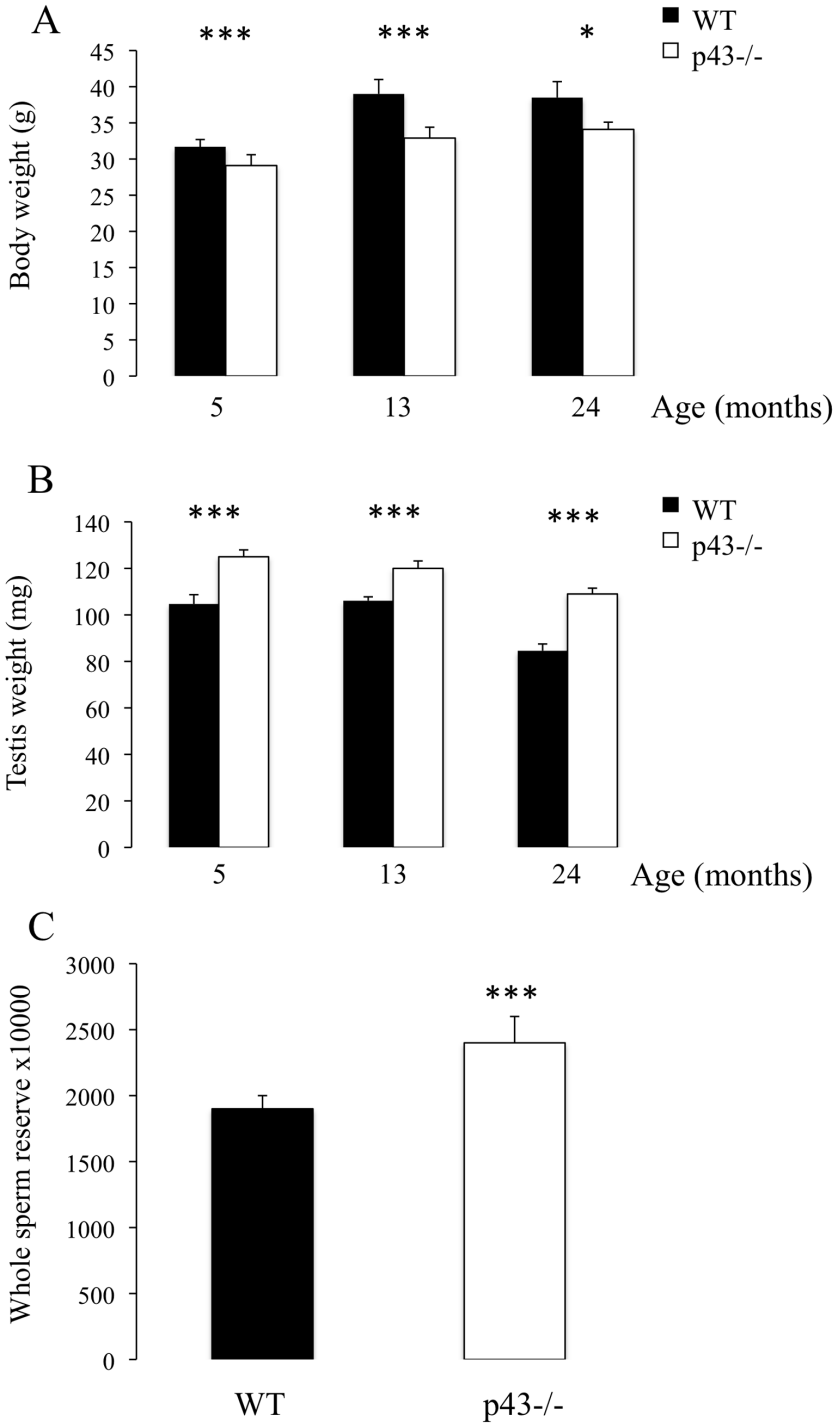
A significant increase in testis weight and whole testicular sperm reserve was observed in p43−/− mice. A) Body weights of mice at 5, 13 and 24 months of age (n = 8 at 13 and 24 months of age, n = 24 at 5 months of age). B) Testis weight of mice at 5, 13 and 24 months of age (n = 8 at 13 and 24 months of age, n = 24 at 5 months of age). C) Whole testicular sperm reserve of mice at 5 months of age (n = 23). Data are shown as the mean+/− SEM and statistical analyses were performed using the t-test; *P<0.05, ***P<0.001.

**Figure 2:**
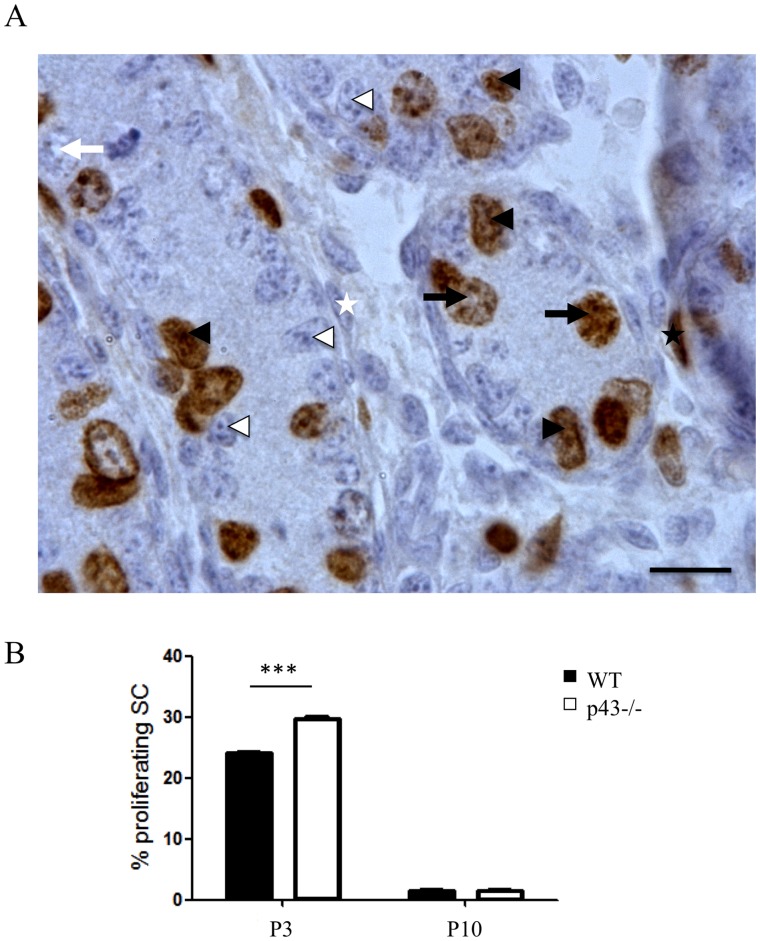
Percentage of *in vivo* proliferating Sertoli cells in p43−/− testis at P3 and P10. A) Immunohistochemical labelling of proliferating cells revealed by BrdU incorporation injected three hours before sacrifice in P3 and P10 testis of p43−/− mice. Arrowheads: Sertoli cells; arrows: germ cells; star: myoid peritubular cells; black: BrdU-positive; white: BrdU-negative. Scale bar 10 µm. B) BrdU negative and positive Sertoli cells were counted and proliferation index was calculated. In p43−/− testis, SC proliferation was increased at P3 in comparison to WT mice (P<0.001). At P10 there is no difference. (n = 3/genotype/age). Data are shown as the mean+/− SEM; statistical analyses; two-way ANOVA followed by Bonferroni’s post-test.

Whereas p43−/− mice displayed a significant decrease in body weight at 5, 13 and 24 months of age in comparison with respective controls (Figure 1A), knock-out animals exhibited a significant increase in testis weight (Figure 1B). At 5 months of age a significant increase in testicular sperm reserve, expressed per testis, was observed in p43−/− mice (Figure 2C). No qualitative histological alterations in seminiferous tubule structures were observed in p43−/− mice, with all germ cell differentiation stages being present. p43−/− males were as fertile as their controls, with no difference in litter size (p43−/−6,9±1.8 pups versus WT 7,2±2.2 pups; n = 30 males per genotype).

In order to investigate a putative effect of p43 deletion on Leydig cell steroidogenic activity, we measured testosterone levels in two conditions: before (basal level) and after (stimulated level) injection of hCG. As expected, injection of hCG induced a significant increase in blood testosterone levels in WT and in p43−/− mice. However, no significant difference between the lines was observed for the basal (WT: 8.6075±5.2285 ng/ml versus p43−/−: 2.38625±0.6725 ng/ml) and stimulated levels (WT: 46.935±7.965 ng/ml versus p43−/−: 50.9825±5.2025 ng/ml). Leydig cell morphology and the surface of the interstitium are similar in p43−/− and WT adult testis.

Altogether our data led us to propose that the increase in testis weight observed in p43−/− mice was probably the consequence of a significant increase in whole testicular sperm reserve (spermatozoa and stage II-VII elongating spermatids).

### The p43 Deletion Increases Sertoli Cell Proliferation in Postnatal Testis

It is well established that the number of SC conditions the efficiency of spermatogenesis as one SC can only support a limited number of germ cells. SC number is defined during foetal and pre-pubertal periods. To investigate if the testicular phenotype of adult p43−/− mice is the result of an increase in the SC proliferation index during post-natal development, we evaluated the proliferation rate of these cells in the young animal (Figure 2A). The percentage of SC incorporating BrdU within 3 hours (Figure 2B) was evaluated in p43−/− mice at P3 and P10 (i.e. when SC proliferation is elevated (P3) and when SC are arresting (P10)). The SC proliferation index was significantly higher in p43−/− testis than in control animals at P3, whereas at P10 it was similar to the controls (Figure 2B). This result shows that SC proliferation in p43−/− mice is mediated in part by p43 during post-natal development. p43 depletion in mice does not affect FSH level (p43−/−: 0.96±0.05 versus WT: 1.03±0.04, ng/100 µl).

### Cell Cycle Gene Expression is Altered in p43−/− Testes at P3

**Figure 3:**
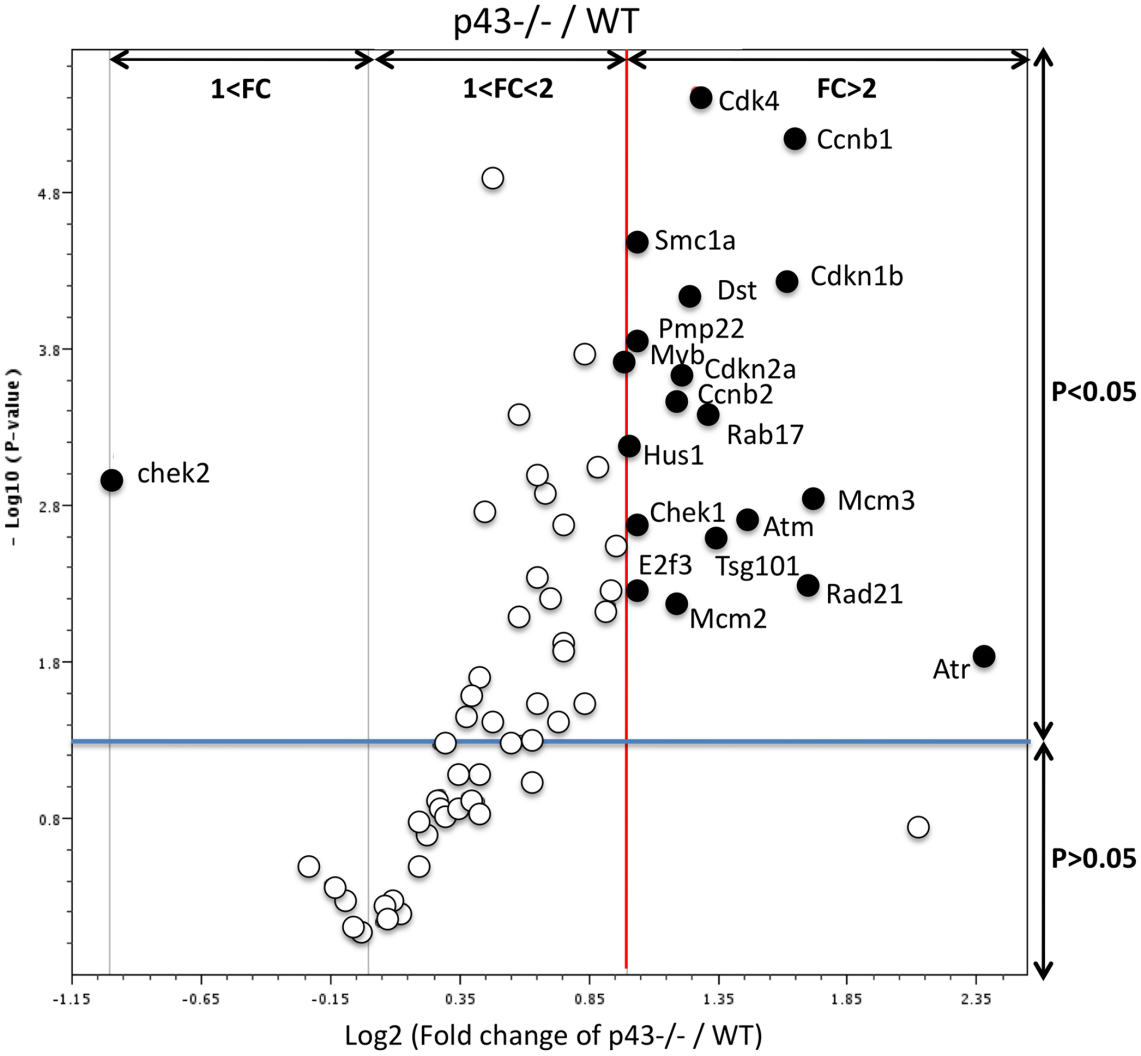
Cell cycle gene expression is impacted in p43−/− testis at P3. The volcano plot is an arbitrary representation (proposed by the web-based RT^2^ prolifer PCR Array Data Analysis program) of the fold change (FC) for each of the 84 genes in the array. It represents the log2 FC of each gene expression between the p43−/− group (n = 6) and the control group (n = 8) versus the negative Log10 P-values from the t-test. The red vertical line indicates that the gene expression fold change threshold is 2. The blue horizontal line indicates that the P-value of the T-test threshold is 0.05. Genes, which were significantly upregulated in p43−/− in comparison with controls, were indicated.

In order to highlight actors in the cell cycle putatively involved in the increase in SC proliferation observed in p43−/− mice at P3, we analyzed 84 candidate genes by Q-PCR (table 1), which are known to be involved in the cell cycle, using the RT2 Profiler PCR Array dedicated to the mouse cell cycle. In p43−/− testis at P3, 19 genes were significantly up-regulated (Figure 3, black circles; Table 1) (2-tailed t-test; p<0.05; main difference equal to or greater than 2-fold). These genes are mainly involved in regulation of cell cycle, the control of transition between the each of the cycle phase, the DNA replication and repair. Several of these genes are cell cycle checkpoints and arrest signals. Chek1 (Checkpoint kinase 1 homolog (S. pombe)) gene which is involved in cell cycle arrest and DNA repair, was the only gene down-regulated (Table 1).

**Table 1:** Cell cycle gene expressions measured by Q-PCR.

Genes Symbols		FC	P-value
Abl1	C-abl oncogene 1, non-receptor tyrosine kinase	ND	−
**Atm**	Ataxia telangiectasia mutated homolog (human)	**2,75**	**0,002**
**Atr**	Ataxia telangiectasia and rad3 related	**5,21**	**0,016**
Aurka	Aurora kinase A	1,27	0,084
Aurkb	Aurora kinase B	1,35	0,125
Bcl2	B-cell leukemia/lymphoma 2	1,58	0,028
Birc5	Baculoviral IAP repeat-containing 5	ND	−
Brca1	Breast cancer 1	ND	−
Brca2	Breast cancer 2	0,98	0,856
Casp3	Caspase 3	ND	−
Ccna1	Cyclin A1	1,03	0,588
Ccna2	Cyclin A2	1,8	0
**Ccnb1**	Cyclin B1	**3,15**	**0**
**Ccnb2**	Cyclin B2	**2,28**	**0**
Ccnc	Cyclin C	ND	−
Ccnd1	Cyclin D1	ND	−
Ccnd2	Cyclin D2	4,4	0,184
Ccnd3	Cyclin D3	1,55	0,004
Ccne1	Cyclin E1	1,62	0,006
Ccnf	Cyclin F	0,84	0,334
Cdc20	Cell division cycle 20 homolog (S. cerevisiae)	ND	−
Cdc25a	Cell division cycle 25 homolog A (S. pombe)	ND	−
Cdc25c	Cell division cycle 25 homolog C (S. pombe)	1,24	0,142
Cdc6	Cell division cycle 6 homolog (S. cerevisiae)	ND	−
Cdc7	Cell division cycle 7 (S. cerevisiae)	1,48	0,008
Cdk1	Cyclin-dependent kinase 1	1,7	0,013
Cdk2	Cyclin-dependent kinase 2	1,95	0,002
**Cdk4**	Cyclin-dependent kinase 4	**2,41**	**0**
Cdk5rap1	CDK5 regulatory subunit associated protein 1	1,1	0,602
Cdk6	Cyclin-dependent kinase 6	1,91	0,006
Cdkn1a	Cyclin-dependent kinase inhibitor 1A (P21)	ND	−
**Cdkn1b**	Cyclin-dependent kinase inhibitor 1B	**3,08**	**0**
**Cdkn2a**	Cyclin-dependent kinase inhibitor 2A	**2,31**	**0**
Cdkn2b	Cyclin-dependent kinase inhibitor 2B	1,35	0,086
Cdkn3	Cyclin-dependent kinase inhibitor 3	ND	−
**Chek1**	Checkpoint kinase 1 homolog (S. pombe)	**2,06**	**0,002**
**Chek2**	CHK2 checkpoint homolog (S. pombe)	**0,51**	**0,001**
Cks1b	CDC28 protein kinase 1b	0,94	0,549
Ddit3	DNA-damage inducible transcript 3	1,07	0,529
**Dst**	Dystonin	**2,36**	**0**
E2f1	E2F transcription factor 1	1,15	0,307
E2f2	E2F transcription factor 2	1,56	0,051
**E2f3**	E2F transcription factor 3	**2,06**	**0,005**
**E2f4**	E2F transcription factor 4	1,32	0,024
	**Genes Symbols**	**FC**	**P-value**
Gadd45a	Growth arrest and DNA-damage-inducible 45 alpha	1,77	0,03
Gpr132	G protein-coupled receptor 132	ND	−
**Hus1**	Hus1 homolog (S. pombe)	**2**	**0**
Itgb1	Integrin beta 1 (fibronectin receptor beta)	1,39	0,038
Mad2l1	MAD2 mitotic arrest deficient-like 1 (yeast)	ND	−
**Mcm2**	Minichromosome maintenance deficient 2 mitotin	**2,28**	**0,006**
**Mcm3**	Minichromosome maintenance deficient 3	**3,35**	**0,001**
Mcm4	Minichromosome maintenance deficient 4 homolog	1,93	0,006
Mdm2	Transformed mouse 3T3 cell double minute 2	ND	−
Mki67	Antigen identified by monoclonal antibody Ki 67	1,67	0,036
Mre11a	Meiotic recombination 11 homolog A (S. cerevisiae)	1,3	0,116
Msh2	MutS homolog 2 (E. coli)	1,26	0,147
**Myb**	Myeloblastosis oncogene	**2**	**0**
Nbn	Nibrin	1,15	0,196
Nek2	NIMA (never in mitosis gene a)-related expressed	ND	−
	kinase 2		
Notch2	Notch gene homolog 2 (Drosophila)	0,96	0,823
Pkd1	Polycystic kidney disease 1 homolog	ND	−
**Pmp22**	Peripheral myelin protein 22	**2,03**	**0**
Ppm1d	Protein phosphatase 1D magnesium-dependent,	1,22	0,11
	delta isoform		
**Rad17**	RAD17 homolog (S. pombe)	**2,48**	**0**
**Rad21**	RAD21 homolog (S. pombe)	**3,26**	**0,004**
Rad51	RAD51 homolog (S. cerevisiae)	1,58	0,001
Rad9	RAD9 homolog (S. pombe)	1,55	0,097
Ran	RAN, member RAS oncogene family	1,5	0
Rb1	Retinoblastoma 1	ND	−
Rbl1	Retinoblastoma-like 1 (p107)	1,7	0,014
Rbl2	Retinoblastoma-like 2	0,9	0,421
**Sfn**	Stratifin	**2,05**	**0,05**
Shc1	Src homology 2 domain-containing transforming	ND	−
	protein C1		
Skp2	S-phase kinase-associated protein 2 (p45)	1,3	0,034
Slfn1	Schlafen 1	1,83	0
**Smc1a**	Structural maintenance of chromosomes 1A	**2,06**	**0,000**
Stag1	Stromal antigen 1	1,29	0,131
Stmn1	Stathmin 1	1,7	0,002
Terf1	Telomeric repeat binding factor 1	1,03	0,727
Tfdp1	Transcription factor Dp 1	1,35	0,021
Trp53	Transformation related protein 53	1,13	0,174
Trp63	Transformation related protein 63	ND	−
**Tsg101**	Tumor susceptibility gene 101	**2,57**	**0,002**
Wee1	WEE 1 homolog 1 (S. pombe)	1,38	0

For each gene: fold values (FC) and p value obtained in p43−/− mice versus respective controls. Significant genes changed in p43−/− mice are in bold. ND: gene expression was no detected.

**Figure 4:**
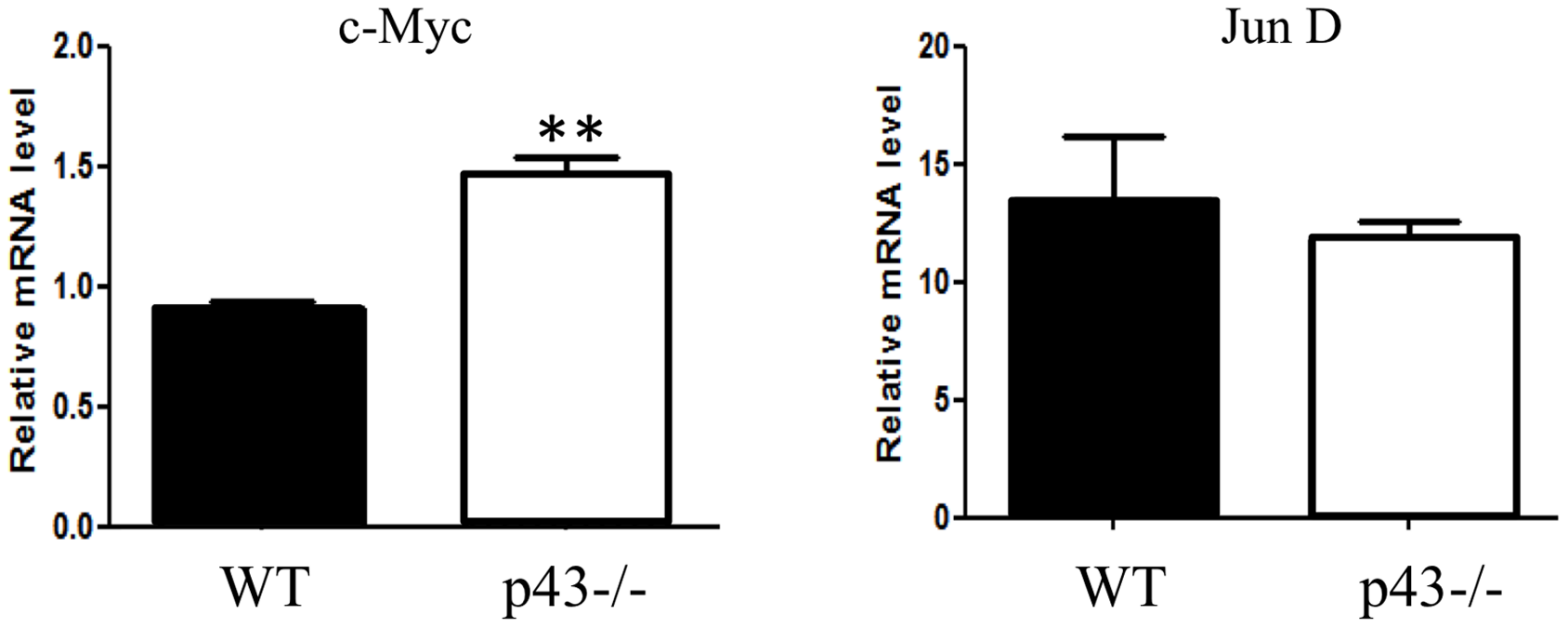
c-myc gene expression is increased in p43−/− testis at P3. Q-PCR analyses for c-myc and junD gene expressions were performed with p43−/− and WT testes at P3 (n = 6 for p43−/−, n = 4 for WT) using specific primers. Normalization was achieved using ß-actin levels. c-myc mRNA levels were increased (P<0,01). JunD mRNA levels were not different in p43−/− and WT testis. Data are shown as the mean+/− SEM and statistical analyses were performed using the student t test.

**Figure 5:**
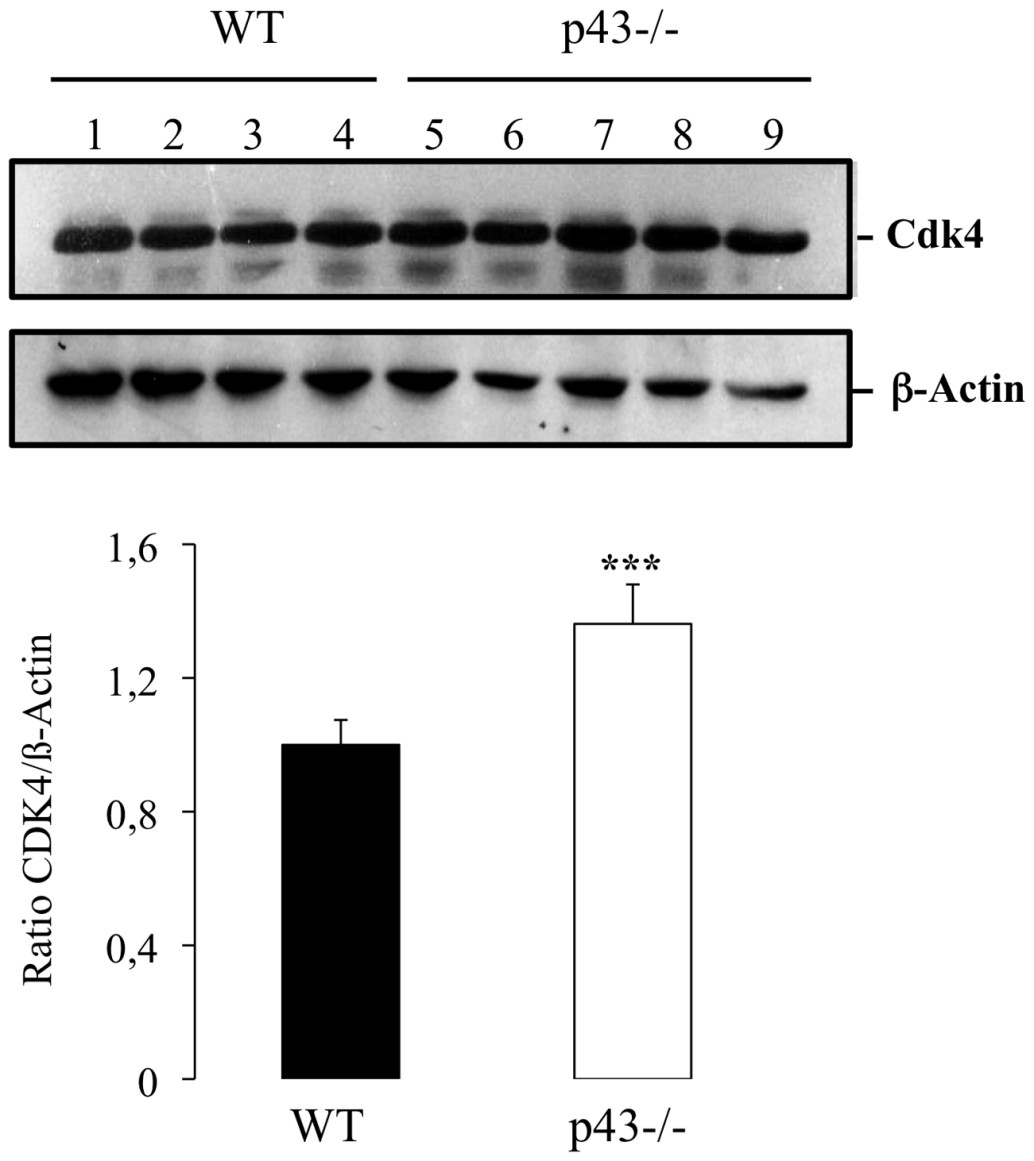
CDK4 expression is increased in P43−/− testis at P3. Western blot analyses were performed with p43−/− and WT proteins extract testes at P3 (n = 5 for P43−/−, n = 4 for WT) using a specific CDK4 and ß-actin antibodies. Normalization was achieved using ß-actin levels. Data are shown as the mean+/− SEM and statistical analyses were performed using the student t test. CDK4 level was increased (P<0,001).

Among the genes up-regulated we found the cyclin cdk4 (2.40 fold). Interestingly, it was previously shown in the TRα^AMI^–SC and TRα0/0 mice lines that T3 negatively controls post-natal Sertoli cell proliferation by activation of TRα1 involving Cdk4/JunD/c-myc pathway [11]. To test the possibility that a similar pathways occurred in p43−/− mice we measured the mRNA levels of c-myc and JunD in p43−/− testis using quantitative RT-PCR with specific primers. We observed a significant increase of c-myc mRNA level in knock-out testis in comparison to controls (1.62 fold, Figure 4) whereas mRNA level of JunD was unchanged (Figure 4). In line with the increase of Cdk4 expression at mRNA levels we showed by western-blot blot analysis a significant raised of the protein in p43−/− testis at P3 (Figure 5).

### The Structure of the Mitochondria in Sertoli Cells is Impaired in p43−/− Mice in Adulthood

**Figure 6:**
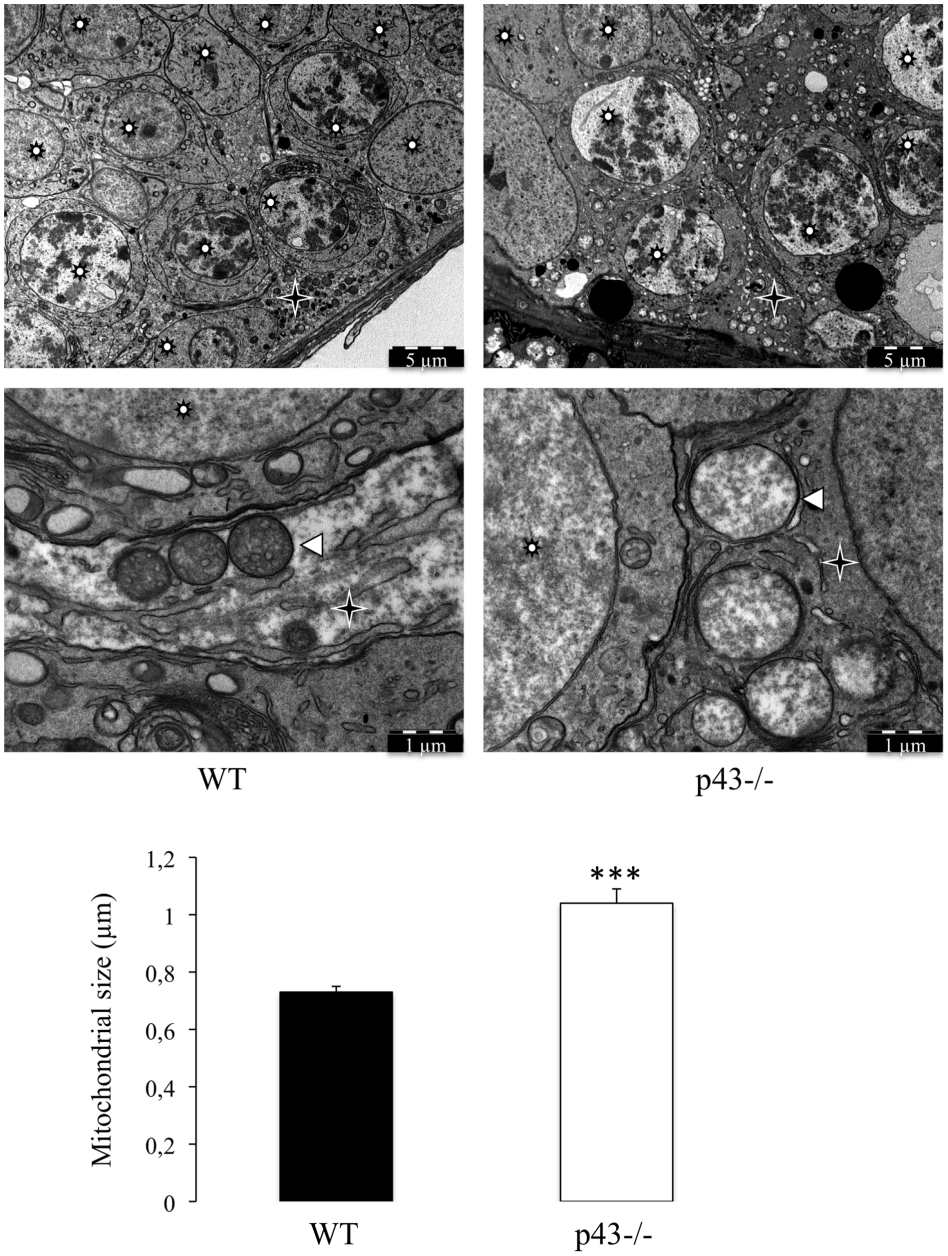
The mitochondrial morphology is strongly affected in Sertoli cells of p43−/− mice. Mitochondria observed in Sertoli cells in the adult testis of wild type mice (WT) and in the adult testis of p43−/− mice at 5 months of age (p43−/−). Mitochondria present in the Sertoli cells of p43−/− mice were morphologically different; they are larger and have a lower opacity to electrons. The mitochondria size was evaluated by measuring the diameter of mitochondria. Data are shown as the mean+/− SEM; statistical analyses; two-way ANOVA followed by Bonferroni’s post-test (n = 50). *** = P<0.001. Star Wheel: Germs cells; star: Sertoli cells; arrowheads: Mitochondria.

**Figure 7:**
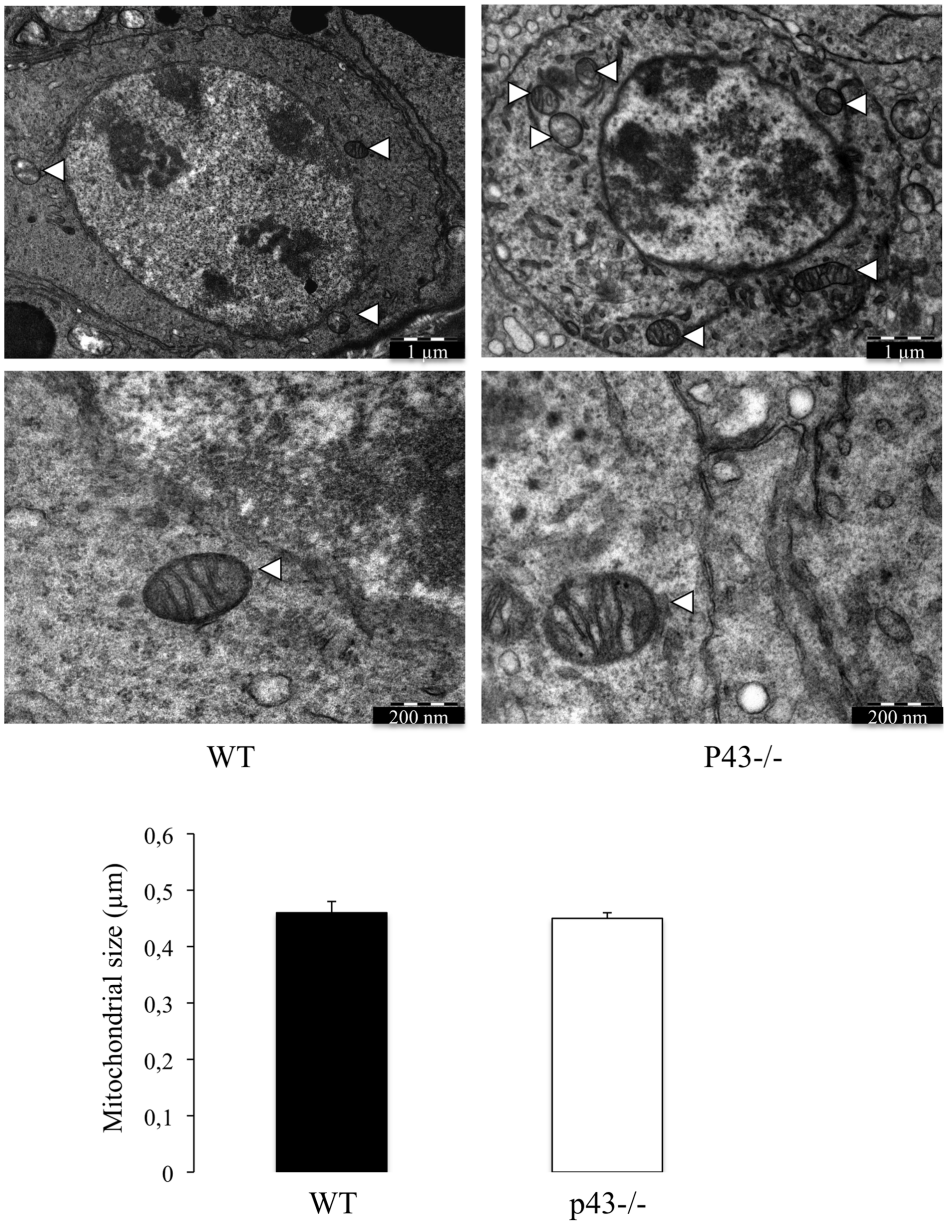
The mitochondrial morphology is unaffected in germ cells of p43−/− mice. Mitochondria observed in germ cells in the adult testis of wild type mice (WT) and in the adult testis of p43−/− mice at 5 months of age (p43−/−). Mitochondria present in germ cells of p43−/− mice were morphological identical at those presenting in germ cells of WT mice. Data are shown as the mean+/− SEM; statistical analyses; two-way ANOVA followed by Bonferroni’s post-test (n = 60). Arrowheads: Mitochondria.

p43 has been shown to control mitochondrial biogenesis in cultured cells [12], [22] and in skeletal muscle [14], [16]. In order to evaluate the influence of p43 deletion on structure of mitochondria in Sertoli and germ cells we performed electron microscopy. In p43−/− mice the shape of mitochondria is only modified in SC (Figure 6). They are more expanded showing a lower electron density compared with control mice. On the other hand, mitochondria observed in p43−/− germ cells shown the same histological picture than in controls (Figure 7). This result indicates that p43 deletion induced a deep modification of mitochondrial morphology in SC.

### Mitochondrial Gene Expression is Altered in p43−/− Testes at P3

**Figure 8:**
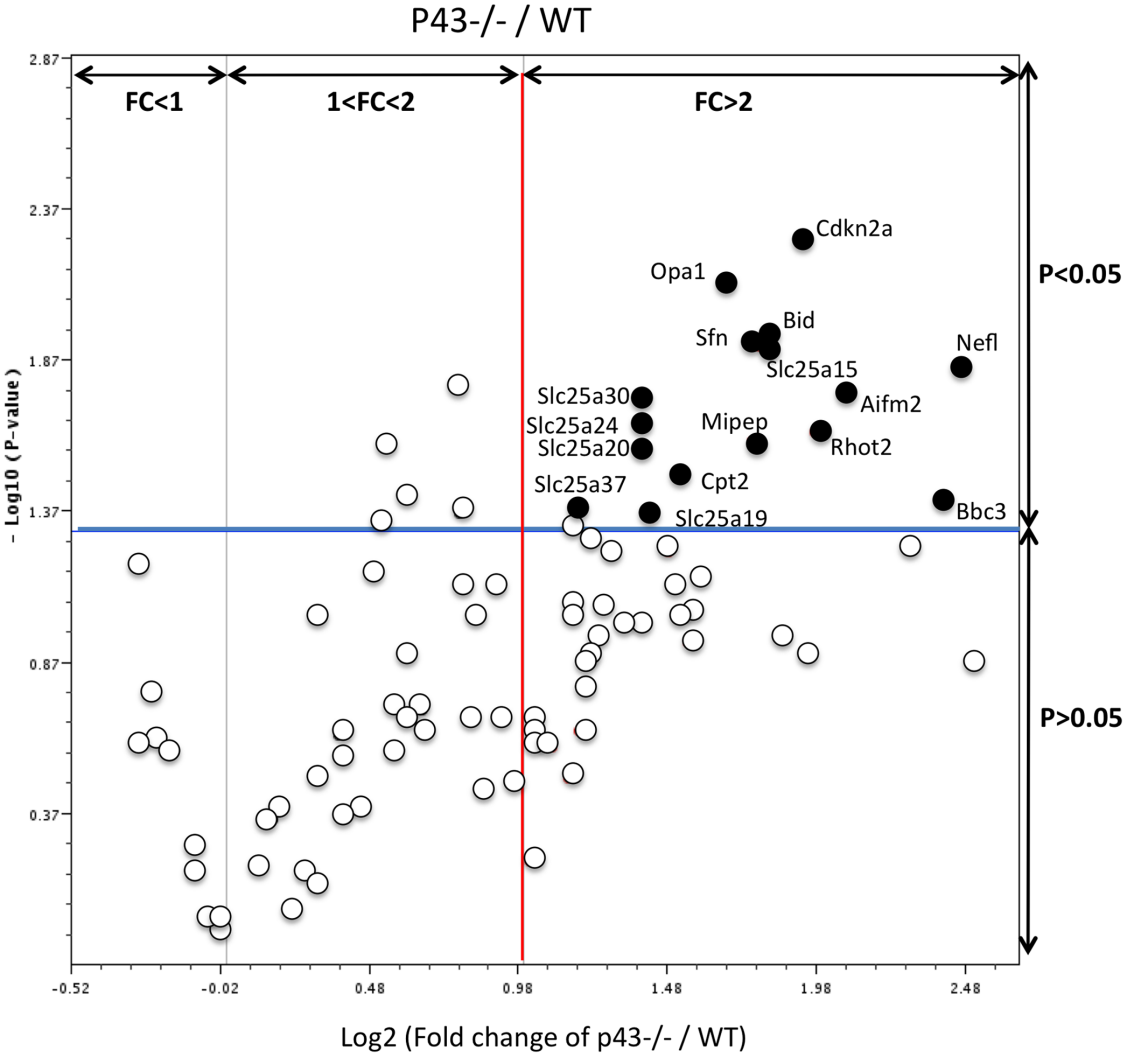
Mitochondrial gene expression is altered in p43−/− testis at P3. The volcano plot is an arbitrary representation (proposed by the web-based RT^2^ prolifer PCR Array Data Analysis program) of the fold change (FC) for each of the 84 genes in the array. It represents the log2C FC of each gene expression between the p43−/− group (n = 6) and the control group (n = 4) versus the negative Log10 P values from the t-test. The red vertical line indicates that the gene expression fold change threshold is 2. The blue horizontal line indicates that the P value of the T-test threshold is 0.05. Genes, which were significantly up-regulated in p43−/− mice in comparison with controls, were indicated.

As previously done for cell cycle actors, we analysed by Q-PCR 84 candidate genes putatively involved in the mitochondrial function which could explain the increase in SC proliferation observed in p43−/− mice at P3, (Table 2). In p43−/− testes at P3, we found 13 genes significantly up-regulated (Figure 8, black circles; Table 2) (2-tailed t-test; p<0.05; main difference equal to or greater than 2-fold). These genes are involved in: small transport of molecules, in import and cleavage of proteins, in metabolism, in localization of proteins mitochondrial localization, in apoptosis and in cell cycle. No down-regulated genes were found (Table 2). These results demonstrate that the mitochondrial T3 receptor plays an important role in many mitochondria functions.

**Table 2:** Mitochondrial gene expressions measured by Q-PCR.

Genes Symbols		FC	P-value
**Aifm2**	Apoptosis-inducing factor, mitochondrion-associated 2	**4.26**	**0.017**
Aip	Aryl-hydrocarbon receptor-interacting protein	2.34	0.127
Bak1	BCL2-antagonist/killer 1	1.74	0.076
**Bbc3**	BCL2 binding component 3	**ND**	−
Bcl2	B-cell leukemia/lymphoma 2	2.05	0.211
Bcl2l1	Bcl2-like 1	2.30	0.158
**Bid**	BH3 interacting domain death agonist	**3.48**	**0.011**
Bnip3	BCL2/adenovirus E1B interacting protein 3	1.31	0.428
**Cdkn2a**	Cyclin-dependent kinase inhibitor 2A	**3.80**	**0.005**
Cox10	COX10 homolog, cytochrome c oxidase assembly protein,	3	0.091
	heme A		
Cox18	COX18 cytochrome c oxidase assembly homolog	1.54	0.208
	(S. cerevisiae)		
Cpt1b	Carnitine palmitoyltransferase 1b, muscle	ND	−
**Cpt2**	Carnitine palmitoyltransferase 2	**2.84**	**0.031**
Dnajc19	DnaJ (Hsp40) homolog, subfamily C, member 19	2.23	0.093
Dnm1l	Dynamin 1-like	2.81	0.074
Fis1	Fission 1 (mitochondrial outer membrane) homolog (yeast)	2.41	0.087
Fxc1	Fractured callus expressed transcript 1	2.27	0.228
Grpel1	GrpE-like 1, mitochondrial	2.51	0.098
Hsp90aa1	Heat shock protein 90, alpha (cytosolic), class A member 1	2.03	0.235
Hspd1	Heat shock protein 1 (chaperonin)	1.81	0.349
Immp1l	IMP1 inner mitochondrial membrane peptidase-like	1.94	0.332
	(S. cerevisiae)		
Immp2l	IMP2 inner mitochondrial membrane peptidase-like	2.13	0.251
	(S. cerevisiae)		
Lrpprc	Leucine-rich PPR-motif containing	2.86	0.094
Mfn1	Mitofusin 1	2.80	0.058
Mfn2	Mitofusin 2	2.31	0.131
**Mipep**	Mitochondrial intermediate peptidase	**3.40**	**0.025**
Msto1	Misato homolog 1 (Drosophila)	2.02	0.617
Mtx2	Metaxin 2	2.34	0.053
**Nefl**	Neurofilament, light polypeptide	**5.56**	**0.014**
**Opa1**	Optic atrophy 1 homolog (human)	**3.19**	**0.007**
Pmaip1	Phorbol-12-myristate-13-acetate-induced protein 1	1.37	0.415
Rhot1	Ras homolog gene family, member T1	1.60	0.228
**Rhot2**	Ras homolog gene family, member T2	**3.93**	**0.023**
**Sfn**	Stratifin	**3.40**	**0.011**
Sh3glb1	SH3-domain GRB2-like B1 (endophilin)	2.22	0.326
Slc25a1	Solute carrier family 25, member 1	ND	−
Slc25a10	Solute carrier family 25 member 10	2.23	0.089
Slc25a12	Solute carrier family 25, member 12	1.75	0.204
Slc25a13	Solute carrier family 25, member 13	2.08	0.240
Slc25a14	Solute carrier family 25, member 14	2.39	0.109
**Slc25a15**	Solute carrier family 25, member 15	**3.46**	**0.012**
Slc25a16	Solute carrier family 25, member 16	2.43	0.057
Slc25a17	Solute carrier family 25, member 17	2.65	0.099
**Slc25a19**	**Solute carrier family 25, member 19**	**2.69**	**0.045**
Slc25a2	Solute carrier family 25, member 2	ND	−
**Slc25a20**	**Solute carrier family 25, member 21**	**2.61**	**0.026**
	**Genes Symbols**	**FC**	**P-value**
Slc25a21	Solute carrier family 25, member 21	ND	−
Slc25a22	Solute carrier family 25, member 23	3.65	0.109
Slc25a23	Solute carrier family 25, member 23	1.49	0.267
**Slc25a24**	Solute carrier family 25, member 24	**2.64**	**0.021**
Slc25a25	Solute carrier family 25, member 25	3.03	0.068
Slc25a27	Solute carrier family 25, member 27	1.77	0.094
Slc25a3	Solute carrier family 25, member 3	2.24	0.057
**Slc25a30**	Solute carrier family 25, member 30	**2.64**	**0.018**
Slc25a31	Solute carrier family 25, member 31	1.52	0.037
Slc25a37	Solute carrier family 25, member 37	2.27	0.042
Slc25a4	Solute carrier family 25, member 4	1.58	0.195
Slc25a5	Solute carrier family 25, member 5	1.16	0.864
Sod1	Superoxide dismutase 1, soluble	1.22	0.659
Sod2	Superoxide dismutase 2, mitochondrial	1.46	0.185
Stard3	START domain containing 3	1.24	0.324
Taz	Tafazzin	1.72	0.043
Timm10	Translocase of inner mitochondrial membrane 10 homolog	1.08	0.654
	(yeast)		
Timm17a	Translocase of inner mitochondrial membrane 17a	1.23	0.730
Timm17b	Translocase of inner mitochondrial membrane 17b	1.53	0.122
Timm22	Translocase of inner mitochondrial membrane 22 homolog	1.30	0.278
	(yeast)		
Timm23	Translocase of inner mitochondrial membrane 23 homolog	0,8	0.256
	(yeast)		
Timm44	Translocase of inner mitochondrial membrane 44	1,4	0.065
Timm50	Translocase of inner mitochondrial membrane 50 homolog	1.45	0.045
	(yeast)		
Timm8a1	Translocase of inner mitochondrial membrane 8 homolog a1	0,8	0.163
	(yeast)		
Timm8b	Translocase of inner mitochondrial membrane 8 homolog b	0,9	0.259
	(yeast)		
Timm9	Translocase of inner mitochondrial membrane 9 homolog	1	0.959
	(yeast)		
Tomm20	Translocase of outer mitochondrial membrane 20 homolog	0,9	0.238
	(yeast)		
Tomm22	Translocase of outer mitochondrial membrane 22 homolog	1	0.986
	(yeast)		
Tomm34	Translocase of outer mitochondrial membrane 34	1.46	0.025
Tomm40	Translocase of outer mitochondrial membrane 40 homolog	1.71	0.016
	(yeast)		
Tomm40l	Translocase of outer mitochondrial membrane 40 homolog-like	1.14	0.401
	(yeast)		
Tomm70a	Translocase of outer mitochondrial membrane 70 homolog A	0,9	0.670
	(yeast)		
Trp53	Transformation related protein 53	0,8	0.064
Tspo	Translocator protein	1.12	0.450
Ucp1	Uncoupling protein 1 (mitochondrial, proton carrier)	ND	0.136
Ucp2	Uncoupling protein 2 (mitochondrial, proton carrier)	1.30	−
Ucp3	Uncoupling protein 3 (mitochondrial, proton carrier)	ND	−

For each gene: fold values (FC) and p value obtained in p43−/− mice versus respective controls. Significant genes changed in p43−/− mice are in bold. ND: gene expression was no detected.

## Discussion

We show for the first time a mitochondrial control of the differentiation of Sertoli cells by T3 via the mitochondrial T3 receptor p43**.** p43−/− mice display a testicular phenotype which is very similar to the phenotype of TRα0/0 knockout mice [10], with an increase in testicular sperm reserve and testis weight. *In vivo* at P3, the SC proliferation index was significantly higher in both p43−/− and TRα0/0 mice than in their respective controls. Recently, it was demonstrated that the dominant-negative TRα1L400R5 (TRα^AMI^) only expressed in Sertoli cells displays a testis phenotype, which is very similar to the phenotype of TRα0/0 and p43−/− mice [11]. These interesting results evidenced that an increase in round spermatid number was the consequence of an increase in the proliferation rate of Sertoli cells during postnatal period. The similar phenotype observed in p43−/−, TRα0/0 and TRα^AMI^–SC testis prompt us to propose that the mitochondrial p43 receptor could be the main T3 receptor isoform involved in the physiological situation of T3-control of the post-natal Sertoli cell development. The proliferation rate of Sertoli cells during post natal period is mainly regulated by FSH. But recently Pitetti *et al.* show that ablation of insulin/IGF signalling reduction in testis size and daily sperm production as a result of a reduced proliferation rate of immature SCs during the late fetal and early neonatal periods. These analyses revealed that the insulin/IGF signalling pathway is required for FSH-mediated SC proliferation [23]. Here we found that the plasma FSH level was the same in p43−/− mice than in WT mice at 5 months of age. However, we have previously showed that the depletion of p43 induces a loss of glucose-stimulated insulin secretion [17]. Insulin levels were significantly higher in p43−/− mice in fasting condition and lower after refeeding. Perhaps, these defects in insulin secretion in p43−/− mice could activate insulin/IGF pathway and potentiate the action of FSH on Sertoli cells.

This result demonstrates and confirms that the mitochondrial p43 receptor has physiological functions. In fact, recently, this receptor has been shown to be involved in the control of the secretion of insulin from the pancreas and glucose homeostasis [17] and to affect muscle mass and the metabolic and contractile features of myofibers in mice [16]. The physiological role of this receptor is also confirmed by our study showing its importance for T3 control of Sertoli cell differentiation during the postnatal period. The same testicular phenotype was observed in p43−/− and TRα^AMI^-SC transgenic mice lines. In p43−/− mice, we are showing that only the mitochondrial isoform p43 receptor is disabled. The TRα1 and 2 nuclear isoform expressions were unaffected. In TRα^AMI^-SC mice, there is coexistence of a wild form of TRα1 and a dominant-negative form, TRα^AMI^. The mitochondrial p43 receptor expression is unaffected (personal communication of Frederic Flamant, creator of the TRα^AMI^ line). Because p43 is a truncated form of TRα1, this implies that in TRα^AMI^-SC mice, the wild form of p43 coexists with a dominant-negative form of the mitochondrial receptor. This result suggests that the dominant-negative form of p43 is able to block the physiological function of p43 in mitochondria.

The Q-PCR array analysis of the expression of a focused panel of genes involved in cell cycle regulation indicates that 19 of the 84 genes tested were significantly up-regulated. These data suggest that p43 through a mitochondrial-nuclear cross-talk regulates the expression of genes involved in the cell cycle in testis as previously described for others cells and tissues [14], [24]–[26].

The comparison of the list of up-regulated genes in the testis at early postnatal period (P3) in p43−/− and in TRα^AMI^-SC mice could provide some clue of the molecular mechanisms underlying the ability of T3 to arrest SC proliferation. Whereas in TRα^AMI^-SC testes, only 4 genes were significantly up-regulated [11], 19 genes were concerned in p43−/− mice. Interestingly, Cdk4, Sfn, and Cdkn2a were up-regulated in both cases. Our data suggest that the Cdk4 pathway could be the central target of the mitochondrial-nuclear cross-talk, controlling the proliferation of Sertoli cells. In line with this possibility, Cdk4−/− mice [27] have growth retardation and reproductive dysfunction associated with testicular atrophy and hypoplastic seminiferous tubules in the testis. Cdk4 transcription is controlled by two main regulators: c-myc (activator; [28], [29]), and JunD (repressor; [30], [31]). In TRα^AMI^-SC, an increase in the expression of c-myc and a decrease in JunD expression were shown [11]. In p43−/− mice, we only observed an increase in c-myc expression while JunD is not altered. c-myc is known to regulate Cdk4 and mitochondrial function [25]. This data suggesting that c-myc is an important target of mitochondrial activity is fully in line with the work of Seyer et al. [26] showing that in myoblast C2C12, an inhibition of mitochondrial activity by chloramphenicol (which mimics a lack of p43) increased c-myc mRNA and protein levels. Conversely, stimulation of mitochondrial activity by overexpression of the T3 mitochondrial receptor (p43) down-regulates c-myc expression. In addition, c-myc overexpression mimicked the influence of mitochondrial activity inhibition on myoblast differentiation. These data suggest that p43 regulates Sertoli cell proliferation by a similar mechanism involving the control of c-myc expression. Moreover, these set of data suggest that the mechanism identified in the control of the differentiation of myoblasts and Sertoli cells could concern numerous other cell types.

The mitochondrial ultrastructure is deeply altered in Sertoli cells of p43−/− mice while the mitochondrial morphology in germ cells is unchanged. This observation suggests that the p43 receptor was probably not expressed in germ cells. In addition, the alteration of mitochondrial morphology observed in p43−/− Sertoli cells is associated with a deregulation of expression of genes which are involved in mitochondrial function. The p43 receptor is known to act as a T3-dependent transcription factor of the mitochondrial genome, which regulates the synthesis of mitochondrial proteins, oxygen consumption and the activities of the complexes of the respiratory chain [12], [13], [22]. In testis, we have measured the expression level of 84 genes involved in various cellular functions of mitochondria including regulators and mediators of mitochondrial molecular transport, metabolites needed for the electron transport chain and oxidative phosphorylation, intrinsic apoptosis pathway genes activated by intracellular damage signalling. We show that many mitochondrial genes were up-regulated in the testes at early postnatal period (P3) of p43−/− mice. These genes mainly code for molecules involved in transport (Slc25 family), metabolism (CPT2, Mipep, Opa1, Rhot2), anti-apoptosis pathway (AIfm2, Bid) and also in cell cycle control (Cdkn2a and Sfn). We can postulate that this up-regulation of the expression of several genes encoding mitochondrial proteins is intended to offset the decrease of mitochondrial activity which probably occurs in Sertoli cells lacking p43.

In summary, these results establish that p43 depletion in mice induced a testicular phenotype very similar to those observed in TRα0/0 [10] and TRα^AMI^–SC mice [11]. This indicates that the mitochondrial p43 receptor is likely the main T3 receptor isoform involved in the physiological situation of T3-control of the post-natal Sertoli cell development. In addition, gene expression studies provide some molecular mechanisms underlying the ability of T3 to arrest Sertoli cell proliferation. In particular, c-myc and Cdk4 could be the central target of the mitochondrial-nuclear crosstalk, controlling the proliferation of Sertoli cells. Given the results previously obtained on myoblasts [25], these data suggest that p43 could regulate proliferation and differentiation of numerous other cell types by a mechanism involving the control of c-myc expression.
